# Antenatal consultation for parents whose child may require admission to neonatal intensive care: a focus group study for media design

**DOI:** 10.1186/s12884-016-0898-8

**Published:** 2016-05-14

**Authors:** Patrick von Hauff, Karen Long, Barbara Taylor, Michael A. van Manen

**Affiliations:** University of Alberta, Edmonton, AB Canada; Northern Alberta Neonatal Program, Edmonton, AB Canada

**Keywords:** Antenatal consultation, Infant, Media design, NICU, Parent

## Abstract

**Background:**

For parents whose child may require admission to a neonatal intensive care unit (NICU), the antenatal consultation is often their first point of contact with the child’s medical team. Consultation challenges health professionals, as parents may be anxious, overwhelmed, or even exhausted by what is and what might occur. Despite consultation being a common practice, there is a paucity of research on how to support practitioners and parents. The purpose of this study was to gain insights into important relational aspects of antenatal consultation that may be used to spur the development of media to support consultation.

**Methods:**

Focus group, as a data collection method, was employed to gather insights about antenatal consultation from a total of 50 hospital staff and 17 NICU parents from a large urban NICU program in western Canada. Qualitative content analysis was applied to the obtained materials to explicate themes that may serve as necessary understandings for media design.

**Results:**

Participating hospital staff and parents expressed their desire for a good antenatal consultation with comments grouped under the following themes: supporting the building of a caring relation; sharing information in conversation; and, preparing for what is to come.

**Conclusions:**

To support the emerging relations of baby, parent, and hospital staff, a good antenatal consultation needs to convey care, understanding, and empathy; create possibilities for open and genuine conversations; and, foster the buildings of respect, confidence, and trust.

## Background

Antenatal consultation is a core activity of specialists in neonatal medicine. The practical intent is for a practitioner and parent to meet prior to the birth of a child so as to contribute to the care of the mother and the newborn [1]. The consulting practitioner is a member of a larger multidisciplinary team who provides care for the newborn. Consults may be requested when decisions need to be made regarding treatment of the mother or child. Alternatively, consults may be conducted as a means to simply provide anticipatory information to parents regarding the care that their child may need. Consults from neonatal specialists are most commonly requested for issues related to maternal or fetal health in cases of impending premature delivery [2].

Antenatal consultation may serve to obtain informed consent; help to relieve parental anxiety; facilitate a multidisciplinary approach; enable elaboration of medical treatment plans; and, support parenting practices such as breastfeeding and holding [1, 3, 4]. There is significant variation, however, in how practitioners convey and document antenatal consultation information [5, 6]. From survey studies it appears that information related to chances of survival, likely medical problems and treatments, and risk for incurring disability are appropriate to be included [2]. As well, expectant families often need the opportunity to express their feelings; to talk about their pregnancy and baby; and, to discuss how they might interact with their baby after birth (touching, holding, and feeding) [2–4, 7]. Factors related to religion, spirituality, hope, and uncertainty are also important [8–10].

There is a lack of literature on how to support practitioners who engage in antenatal consultations. It is not just that the medical information of the consults is complex—psychosocial issues related to cultural difference, mental health, and teenage parenting are also often at play [11–13]. The antenatal consult is usually the parent’s first point of contact with the neonatal team dealing with ethically challenging and morally difficult predicaments, such as extreme prematurity and complex congenital anomalies [14]. A disease or medical focused model may not be the most appropriate means for antenatal consultation [15]. There is a dearth of support material to facilitate parent-clinician antenatal encounters. As such, there is a potential need for the design and development of media to support antenatal consultation counseling [16].

## Methods

### Aims

The purpose of this study was to gain insights into important relational aspects of antenatal consultation that may be used to spur the development of media to support consultation. We approached healthcare professionals (HCP) and NICU parents as expert sources for the determination of what constitutes good antenatal consultation from a relational ethics perspective to develop guiding principles for media design (design imperatives).

Relational ethics assumes that ethical practice takes place within the context of human relationships, and that human flourishing is enhanced by healthy and ethically sound relationships [17]. In the context of neonatal-perinatal medicine, the newborn infant or developing child is placed at the center of the relational world of the family and hospital staff [18]. Thus from a relational ethics perspective, we need to consider the baby, the family, and hospital staff for understanding good antenatal consultation.

Media may be understood to include any medium or artifact of communication such as patient-family education pamphlets, screen or tablet-based applications, and staff resource websites. A broad conceptualization of media offers varied yet concrete possibilities for imagining creative technologies to support antenatal consultation.

### Data collection

Focus group, as a data collection method, was employed to gather ideas and insights about a range of perceptions, attitudes, and opinions from individuals, expert in antenatal consultation [19, 20]. Focus group method has the advantage of engaging, prompting, and sharing discussions between individuals to formulate fresh perspectives, to question taken-for-granted attitudes, and to clarify existing opinions [21, 22]. Some limitations of focus group method relate to individual disclosure, group dynamics, and organizational issues [22].

A relational ethics framework was used to articulate focus group guiding questions around considerations for the baby, the family, and hospital staff as starting points for antenatal consultation. Guiding questions were as follows: (1) What do we want from antenatal consultation? (2) What are key aspects to the experience of antenatal consultation? (3) How ought we to consider the baby, the parent, and hospital staff in antenatal consultation? (4) What are five wishes of what “it” could and should be? and, (5) What are five wishes of what “it” could not and should not be? The “it” of these last questions served to direct the discussion to design considerations rather than focus on proposing particular media that may or may not be useful. In other words, the focus of these questions was not so much to obtain examples of particular media; but instead, to explore what media should support in an antenatal consultation.

The site of this study was a large urban NICU program in western Canada. We invited hospital staff and parents who currently or previously had a child cared for in the NICU to participate in the focus groups. Hospital staff and parent focus groups were held separately given the literature reporting that parents and staff may have differing perspectives on antenatal consultation, as well as recognizing that both parents and staff might feel uncomfortable speaking openly in front of each other regarding current program issues with antenatal consults [3, 23, 24]. We did not make a priori decisions regarding number of participants. Hospital staff were recruited by e-mails and posters. Parents who had previously had their child cared for in an NICU were recruited by e-mails through the Family Advisory Care Team email directory. Parents who currently had their child admitted to the NICU were invited to participate through a research nurse intermediary. The research nurse only approached families who were felt by the social worker to not currently be under psychosocial duress. At the time of the study, media were not routinely used in antenatal consultations.

In total, we conducted 8 hospital staff focus groups with a total of 50 hospital staff: 15 nurses, 8 respiratory therapists, 6 neonatal trainees, 5 neonatologists, 4 social workers, 3 neonatal nurse practitioners, 2 dieticians, 3 administrative staff, 2 pharmacists, 1 clinical ethicist, and 1 family centered care coordinator. As well, we conducted 4 NICU parent focus groups with a total of 17 parents (13 mothers and 4 fathers). Admitting diagnoses of their children included: prematurity, birth asphyxia, and congenital anomalies.

Staff and senior students from the Department of Art and Design facilitated the focus group sessions. Facilitators received a brief introduction to antenatal consultation prior to the session. The facilitators were instructed to adopt the role of a learner to the process of antenatal consultation and of a novice to the practices of neonatal intensive care. This neophyte role encouraged the facilitators to ask everyday questions, to spur conversation, and to create opportunities for interaction by all members of the group. At the same time, facilitators were able to bring the groups back to focus on the above guiding questions if the discussion drifted significantly away from antenatal consultation. Each focus group was situated around a table with large sketchpads in the center area. All participants were invited to write on the pad if they wanted to express ideas in text. Word prompts in keeping with the relational ethics framework were laid on the table: (1) consider the baby; (2) consider the parent; and, (3) consider the hospital staff. The senior investigator who has experience in both clinical neonatology and qualitative research principally coordinated all focus groups. Focus group sessions lasted approximately one hour. At the close of each session, participants were invited to take and mail in anonymous postage cards stamped with the above word prompts if they wanted to express additional ideas. The senior investigator held debriefings with the facilitators. Data were digitally recorded and transcribed verbatim in preparation for analysis.

### Data analysis

Qualitative content analysis was applied to the materials obtained from the focus groups to explicate themes [25]. This method was chosen with the aim of creating a summary of the data. Articulating themes and subthemes also served the purpose of drawing out the multiple relational subtleties of a good antenatal consultation for the reader. Materials for analysis included: (1) written comments on the participant sketchpads; (2) audio recordings and written transcription of focus groups; and, (3) returned cards. Since few prompt cards were returned, the majority of the data for analysis were from the sketchpads and focus group recordings.

In reviewing the collected materials, the project team focused on the contextual meanings of the speech and text [26]. Themes were developed from a prolonged interpretive engagement with the collected materials, reviewing and re-reviewing all of the data [27]. Since the method of recruitment was in part based on convenience, and thus not necessarily representative of all hospital staff or NICU parents, no weight was given to the frequency of particular identified themes.

After generating various themes and interpretive text, the focus group materials was reviewed again to ensure that the themes spoke to the content of focus group materials. Sampling was declared complete after having included a breadth of participants, and a redundancy of themes among focus groups. Anonymized quotes from the focus group transcriptions were included as evidence for the validity of themes, and to help convey contextual meaning to the reader.

## Results

Participating hospital staff and parents expressed their desire for a good antenatal consultation wherein the “right thing” was done. (Parent) For parents and hospital staff, antenatal consultation is more than a service, “it’s a right” for parents and crucial for them to make “good decisions” for their children. (HCP; Parent) As one staff described: “Every high risk family deserves to have an antenatal consult to talk to somebody before that baby is delivered.” (HCP) The following themes speak to what was considered good antenatal consultation from a relational ethics perspective, and therefore considerations for future media design.

### Building a caring relation

#### First meeting

An antenatal consultation is often the first meeting of the newborn’s medical team and the expectant parents. While the medical team will have an understanding of the clinical issues prompting the consult such as threatened premature labor or suspicion of congenital anomaly, the parents and HCP are new to each other.

“What is the least that I want out of a consult? Just meeting the parents. Even if there’s no time to discuss plans or anything like that. At least if I just meet those parents and they have met me, they’ve seen my face. I think that in itself builds a relationship.” (HCP)

“Having someone come and explain things to me, and even just telling me there’s an NICU here, this is where your baby is going to go. It was great. It really was. I was probably a deer in a headlight at that moment. Like I don’t really remember a lot of what we talked about . . . I just felt, you know, my family is being taken care of.” (Parent)

#### Foundation for confidence and trust

Both hospital staff and parents express that an antenatal consultation affords them with the opportunity to lay the foundations of a relationship:

“For them to have confidence in the team. I guess that’s one of the things I would want from a consultation is that we’re able to develop enough of a relationship so that they have confidence in our team, that we’re providing the best care that we can.” (HCP)

The confidence of a caring relation expresses more than acknowledgement of competence. It requires aspects of respect and caring so that the team can “begin to earn the trust of the parents.” (HCP)

#### Respecting relationships

From this first meeting, hospital staff and parents express a need for an antenatal consult to set the stage for them to provide care for the expectant baby. Setting the stage in a consultation is an introduction not simply to what NICU care might look like for a given baby; but rather to the kind of relationship that parents and hospital staff hopefully will have whereby parental input is respected: “To feel like your opinion as a parent is valued and wanted.” (Parent)

“I think it’s important for parents to get a sense of what they can do. To be empowered, in that situation, even though they probably don’t have any control over many things. What we can do to is validate their role as parents.” (HCP)

From this perspective, an antenatal consult needs a dialogic and supportive atmosphere to situate discussions of parenting between hospital staff and parents. The focus of an antenatal consultation is on a parent’s child as well as the parent’s situatedness so that the parent can understand things and feel understood.

“The consult really should address how to care for your baby while it’s still inside of you, and how do you care for your baby once it gets into the NICU, and things that you can do for your baby to still feel like a parent even though you have zero control over the whole situation.” (Parent)

#### Awareness of asymmetry

There is a particular asymmetry to the parent-hospital staff relationship in antenatal consultation. Prior to putting a face to a name, the hospital staff have reviewed clinical records and are privilege to a family’s medical and social history. Coupled with expert medical knowledge, and often the emotional vulnerability of a hospitalized parent, staff members are positioned authoritatively.

“A consultation is no different than a teaching session for a new resident or student, in that you have to first come to a common understanding of what the goals or objectives are. Then you’ve got to have some kind of activity. And last, there’s some kind of closure or assessment or a plan to see again, or this is a summary of what is going to happen.”(HCP)

For some health professionals, an antenatal consultation is an explanatory rather than conversational activity affording the opportunity to convey necessary information to inform the family. While there is a need for education, there is a potential for the tone of antenatal consultation to be shifted. For example, the nature of the relation of antenatal consultation might become one of competing agendas endangering open dialogue.

“Parents might have already searched on that particular problem and finding, and they might be, like, I have these questions, but the doctor will have their own agenda, their own points. My consult was the doctor’s agenda. I didn’t get a chance.” (Parent)

#### Potential for intrusion

It is not just the building of a caring relation between parent and professional that may be obstructed, hospital staff ought also to be keenly aware that an antenatal consultation has the potential to intrude on the relation between parent and child.

“We have so little choice in our baby’s life when they’re born. If we make a decision, if it’s a decision we can make, just let us make it. [The doctors] know everything. They’ve seen all of your ultrasounds. You’ve had how many examinations. And like we had her name completely picked out, and they said, ‘Yeah, so what’s the name?’ And I said, ‘We have it. We’re not telling anyone about it.’ And luckily they said, ‘Okay,’ and they kind of moved on from that. [Her name] was the one thing that was still private.” (Parent)

While an antenatal consultation is an opportunity for the building of a caring relation between staff with child, it in itself does not entitle the health practitioner to be wholly within the relation of parent and child. Although some parents want to share their expectant child’s name, others might want to keep such personal information private. Health professionals can still show interest and compassion while limiting their intrusion on the parent-child relation.

“When I go in I ask the mom or dad, is the gender of the baby a secret or a surprise or if they know. If they say it’s a surprise, they don’t want to reveal it, so I just refer to the baby as the little one. If they say, ‘Oh, it’s a boy,’ so then I ask do you have a name chosen and I can call him by that name, Gavin, whatever, so then little Gavin, when he comes out he needs, we can support that kind of plan so that’s . . . and parents often ask me ‘So my baby will be taken to NICU. Will I be able to hold him, touch him?’ So they’re showing their emotions, their love. That has to be respected.” (HCP)

#### Connection and community

A challenge for the consultant is maintaining a relationship with the family while being part of a larger specialized team with varied and sometimes competing responsibilities.

“It was nice to meet the medical team . . . although the neonatologist we met, I can’t remember his name, he was never here. I understand that they work in different hospitals. But just to feel that there’s this doctor here who talked to us but he was never here . . . You feel like you’re lacking that potential connection with the doctor.” (Parent)

As parents might yearn for a rapport with a particular professional who comes to know their personal circumstances, such a relationship might be difficult to continue in a healthcare system that lacks continuity in care. As such, the consultant is faced with the challenge of creating a sense that this connection will continue with future healthcare team members. After all, many different nurses, respiratory therapists, dieticians, social workers, and other hospital staff could foreseeably be involved in a family’s care.

Antenatal consultation creates the opportunity to not just build a relation with NICU staff but also with the larger community of current and graduate NICU families.

“The experiences of other parents would be helpful to have access to. Just talk to them so you don’t necessarily feel so alone. Connecting . . . so that you can just know that there are other parents with babies in the world who have lived and survived and thrived with having these complications.” (Parent)

### Sharing information in conversation

#### Gathering and transferring information

A good antenatal consultation involves the gathering and providing of information by HCP and expectant parents.

“It’s a two-way street. We have to find out from them what they would like for this child. And they need to find out what we expect clinically or at least about what we think the child’s going to be faced with.” (HCP)

Parents and health practitioners express repeatedly that information needs to be realistic and understandable, the “full picture.” (Parent)

“They need to have all the information so they can make the right decision for their baby, for their family, because it’s going to affect their whole family.” (HCP)

#### The weight of information

Antenatal consultation has the potential of overwhelming families who might already be anxious and exhausted. Each piece of information discussed in the consult has weight and may cloud other information. More so, the information is often coming at a stressful time, making it hard to grasp and remember what was said. The HCP needs to be sensitive to the meaning of different kinds of information and to the situational stresses of the expectant parent.

“There was a lot of information and overwhelming information, yeah. And sometimes the things you hear are really imperative. Like changing to your future. And you sometimes focus on that. Like, oh my god, she could be blind . . . and then everything else is kind of a fog after.” (Parent)

#### Challenge of consistency

From health professionals, there is a concern that consistent information is provided in consultation.

“Consistency of the information, consistency of site-based stats, consistency on our own policies and practices . . . We just want the same information to everybody so we can reinforce what the parents have been told.” (HCP)

Bias speaks to the concern that individual health practitioners might give information in such a manner that they systematically direct parents to particular choices at the expense of consideration for other choices. It does not simply result in what information is given: “It is also in the way the doctor explains it, and how the parent hears it.” (HCP)

“I found that everything got sugar coated in my consult. And it was quite frustrating when you actually figure out what reality is.” (Parent)

“I think sometimes, like, and it’s very hard because all babies are different and every situation is quite different but almost like some consistency, because I feel like a lot of the times, depending on who is going down for the consult, you can get a totally different plan and it can be the exact same situation.” (HCP)

Consistency is challenging because antenatal consults do need to be individualized to particular situations with tensions between giving the right amount of information while being sensitive to situational constraints and stresses. Still, inconsistency in provision of information might also hurt relationships: “The impact of two slightly different messages, both potentially very reasonable, can confuse patients and lower trust.” (HCP)

#### Conversation openings

Antenatal consultation is not simply an activity of information transfer—a talking at one another or an asking and answering of questions. Communication requires “honest and open conversation” (HCP), or as one parent describes “organic conversation.” (Parent) A good antenatal consultation carries the potential for conversation as it creates openings for parents and hospital staff to talk together from a common ground. Given antenatal consultations are usually first meetings about issues that parents might not have ever considered, it might be hard to start a conversation.

“Most of the time parents don’t know what to ask . . . Some parents might have more specific questions and some parents just have no clue where to start.” (HCP)

Parents might need “preparation” or “help” to know what types of questions they can and should ask, “just to start the conversation.” (Parent) It is not simply that parents need education regarding medical language or knowledge, but rather a sense of the potential conversation of antenatal consultation so they can enter in a shared dialogue that is appropriate to their situation. The talk in a conversation involves empathy and compassion, “being there,” for the purpose of the talk rather than simply being there to arrive at a decision or a plan. (Parent)

#### Conversation points

There are many possibilities for conversations that might be appropriate for a given situation.

“And I guess it depends on how much you can take in at that time too. But you know, explain the ventilator, the CPAP, and things like that. But again, I don’t know if at that time, that’s what you’re worried about. So it might be easy for me to say right now but at that time, I might not. If they start talking to me about a CPAP, I might not care. Because all I really care about is this baby going to be okay? But I think visually just to show you things that they can do to help the baby survive.” (Parent)

“It depends on the person. I mean I like hearing statistics too but sometimes it’s scary to hear those things. Some people might like it, and some people might not like to hear those things.” (Parent)

For some parents, statistics relating to “survival” and “any permanent issues that the child could have” are crucial conversation points for making decisions. (Parent) Yet conversations do need to be individualized: “Because everyone will have a different right time for certain conversations.” (Parent)

“Giving us stats of our baby’s survival rate doesn’t matter when you decide to have your baby . . . It’s irrelevant. Once you decide to have your baby, you decided to have your baby. Respect the decision and move on.” (Parent)

#### Respecting individuality

Following a set script for antenatal consultation might inadvertently alienate or disrespect families. Information can only be appropriately tailored to a particular situation if conversation openings are created and also respectfully closed when families come to particular decisions. Creating some and closing other conversation openings might reflect bias— an inclination to present or hold a partial perspective—on the part of the practitioner.

“And respecting that baby, that this is a baby that is wanted, that will be well taken care of. And yes, of course, we want everything done possible to help this baby. Yeah. And there’s a difference between letting you know what your options are as opposed to this is what you should do.” (Parent)

The possibility for a particular “conversation to unfold should be there for any parent if they want to talk about it.” (Parent) Following, “different parents need different resources.” (Parent) As one parent said, “I think the best thing is to have them there and available. And when you’re ready, you’ll look at them.” (Parent)

#### Professional obligations

HCP do express their obligation to inform parents of particular information whether the parents “know they need to hear it or not” for decision making and so the parents “understand what the problem is.” (HCP)

Antenatal consultation, as an activity of conversation, brings decision making to a shared activity of health professional and expectant parent. A decision is not simply a finished product, but must be appreciated in its process of becoming. Conceptually, conversation allows for negotiated decisions wherein both parent and staff are involved. The emphasis should be on the process of decision making, rather than merely on agreeing what option to select from an expertise model. Failing to create openings for conversation makes for a poor antenatal consultation, and yet, health professionals and parents do struggle with the question of to what extent health professionals should give their own opinion. Clearly such personal opinion information ought to be owned by the HCP and not pressed on a parent.

“Definitely the parents should have the opportunity to hear the physician’s honest opinion. And I’m not sure, but it may be beneficial for the physician to give that opinion regardless. Medical and personal. I think both would be useful.” (HCP)

“Trust to give you the information so you can make the right decision for you personally, no matter what it is. That as a parent, you’re the one living with the decision, not the doctors. And your child. Your child is living with the decision and you’re living with the decision.” (Parent)

### Preparing for what is to come

#### Preplanning in anticipation

An antenatal consultation is undertaken in anticipation of the birth of a child, and the necessity that a team of people will need to be involved in the care of that child: “it sets up a plan of action.” (HCP)

“It’s a preplanning thing for the unit, for the nursing staff, the respiratory therapists, the dieticians, for everybody to sort of know what the expectations are so we can be prepared.” (HCP)

A good consult includes “a clear plan for the whole team, for the medical team and the family, to keep everyone together in working towards the well-being of the child.” (HCP) The plan should be able to “evolve and carry through a baby’s stay.” (HCP)

A plan developed during an antenatal consult contains more than pragmatic steps, it also conveys a “basic sense” of things to come: “what you could expect” and “who you can expect to see caring for your child.” (Parents)

“I would have been out of my mind if I didn’t actually have any sort of knowledge coming into this. Like if they weren’t able to come and see me, and all of a sudden they take her and they go . . .” (Parent)

The plan fosters caring relations, so that the family can trust that the staff has their entire family’s best interest at heart, and also to foster the possibility for the parent to become part of the team caring for the child. Professionals have a responsibility to engage with expectant mothers and fathers as parents: to express a genuine concern for the family as a whole.

#### Uncertainty and possibilities

Uncertainty clouds the prognostication and planning for the child and family: “Even with the most update information we have, things can change quickly.” (HCP) In a good consult, health practitioners and families have conversations about “possibilities” for the particular baby and his or her family. The plan of possibilities may thus be easier to work with if it expresses hopes and values instead of promises and commitments.

“Our whole consult was wrong. Everything they said was wrong, the entire thing from beginning to end . . . I know you don’t want to ever treat it like worst case scenario, worse than it is. But I think you just need to know what you could expect.” (Parent)

“I asked, ‘Will I be able to hold my baby?’ They insistent, ‘Oh, yes, of course. Yeah, you'll totally get to cuddle with her. She should stay there for about 45 minutes.’ Then I had her and they’re like, ‘Oh, no, we need to get her over now because we don’t know what’s going on.’ So I didn’t get to hold her. Just don’t make empty promises. They should have said, ‘We would like for you to be able to [hold her] but we can’t guarantee it.’” (Parent)

Parents need a sense of both what will likely happen and what might occur. In part, these are prerequisites for informed consent. Existentially, these happenings are formative for possible journeys, paths, or experiences for NICU families. Discussing possibilities with parents also necessitates discussing the kind of support that can be provided.

“There will be a multi-disciplinary team that will look after you and it doesn’t need to get in to specifics . . . but there are pharmacists, there are dieticians, there are social workers, respiratory therapists, it’s very comprehensive who will look after you and your child.” (HCP)

#### Sharing the planning and plan

Parents need to “feel empowered” and “involved” in developing a plan for care recognizing that each situation is unique.

“By understanding parent’s burden, try to turn every single decision into a shared decision. Precisely in burden-laden decision making, that you know the team can figure out what does this family need to own of this decision, because that’s the one thing you can’t really generalize. Some families want it to be the doctor’s decision, some families need it to be their decision, and getting that right is tough.” (HCP)

Given that the provision of neonatal care is a team effort, the antenatal consult does need to be shared back with members of the larger team who do not attend the consult to promote understanding, transparency, and consistency.

“From a bedside staff perspective, probably there isn’t enough communication as to what was discussed antenatally so that the people can be comfortable who are caring for the baby postnatally, so that they know the situation that the family’s coming from and what the parents’ feelings truly are ‘cause there is a lot of conflict at times there.” (HCP)

While consistency can come from a plan itself, meaning children of similar situations being treated similarly, consistency in the case of decision making for complex situations such as resuscitation at the cusp of viability, requires consistency in the way consultation conversations are handled. Staff need to feel confident in the process of antenatal consultation and have a secure sense of the situation at hand. They need to know what was discussed and how that discussion will impact the care that they will provide for the expectant parents and newborn child. There is clearly an unspoken minimum standard as to what an antenatal consultation should cover.

#### Respecting hope with uncertain outcomes

A good antenatal consultation recognizes the inherent uncertainty of neonatal intensive care. In such situations, both staff and families do need support in caring for premature or unwell infants to hope for good outcomes yet also deal with challenging possibilities.

“And how to balance, right, that information by allowing them to retain, you know, their hope that this child is doing well and be realistic about what we think the possible outcome’s going to be, given that it’s really unknown. You know, we don’t have the right to take away hope at any given point to a family.” (HCP)

The plan of antenatal consultation does not simply disappear at the closure of the consultation. The plan stays with the child-family whether they await subsequent consultations or delivery of a baby requiring NICU care. While it may be impossible for the plan to be entirely fixed or rigid, the consult ought to offer clarity and guide how to proceed when the child is born. A plan expresses an intention to embark on a particular path recognizing that different possibilities exist. Ultimately, even the best antenatal consultations still necessitate ongoing postnatal consultations as conversations between HCP and families.

## Discussion

The themes of supporting the building of a caring relation; sharing information in conversation; and, preparing for what is to come may be conceptualized as necessary understandings for media design that support good antenatal consultation from a relational ethics perspective.

From the perspective of supporting the building of a caring relation, media should support the emerging relations of baby, parent, and hospital staff rather than seek to supplant them. This is particularly true for the relation of parent and child given that an antenatal consultation carries the capacity to result in first parenting decisions [28]. For example, print media proceeding or following antenatal consultation ought to be designed so it introduces caring relation, and opportunities for interacting as such, between health professionals and parents both during antenatal consultation and within the NICU. In comparison, to support the relation of parent and child, materials should introduce the possibility for having parenting discussions. Placing relations at the forefront of media design responds to the existent medical literature that evidences the importance of health professional and patient-family relations [29, 30].

It is clear from this study that designers of media should be understanding of possible family experiences of medically complicated pregnancies. Media needs to be flexible and accommodate different family situations, and potentially even promote reflection around individual, family, and cultural values that might be important in making parenting decisions. Given that physicians seem to experience difficulty in identifying parents’ expectations and wishes from antenatal consultation [16], media should be designed to facilitate parental input into the various directions that an antenatal consultation might take.

Realizing antenatal consultation as an activity of conversation reveals that antenatal consultation ought to entail more than explanations or other goal-directed language activity. Genuine conversation involves aspects of listening to appreciate meanings holistically [31]. Although it might seem challenging in the context of a high acuity or emergent antenatal consultations to focus foremost on understanding patient-families experiences, this action reflects ethical practice: to be there for patient-families. From a conversational relation, a practitioner may be sensitive to the ethical particularities of a situation so that he or she can elicit and respond to a parent’s concerns: Will my child survive? Will my child be all right? What will my child look like? When can I hold him? What will life be like for us? Conversations as relational spaces are ultimately necessary for shared decision making to relieve families of the inherent burden of difficult clinical decisions yet still avoid a paternalistic paradigm [23, 24].

While it might come naturally for some health professionals and families to enter the mutual space of conversation, others might benefit from primes, prompts, or supports to engage in consultation conversation. From a design perspective, lists of topics that parents often find helpful to discuss in antenatal consultations might be provided in advance of consultations so parents are readied for conversation. In comparison, images of parenting in the NICU might be used during antenatal consultation as conversation points, stirring parents to identify and discuss issues or concerns that might be meaningful for them. Providing topics or images for potential conversation is quite different from a prescriptive checklist that seeks to standardize the content of antenatal consultation to a script; taking away the capacity for parents and health professionals to dwell in conversations most appropriate for their given situations. Conversation points are also quite different from prepared educational modules to be viewed and learned from in isolation from the contact of conversation.

Media has the potential of attenuating bias by offering standard topics or openings for conversation, by including consistent information for practitioners to communicate and by creating opportunities for practitioners to present information in more than one way [32, 33]. Communication in antenatal consultation is inherently demanding such that HCP might benefit from media tools to help ask and answer questions [16]. Such tools need to be commensurate to a variety of clinical and patient-family factors such as impending delivery, limited parental education, and varying cultural expectations. As such, media ought to be designed to promote understanding both seemingly basic and clearly complex information in time limited and more deliberative situations. Foreseeably, providing materials before, during, or after antenatal consultation creates additional opportunities for parental reflection and understanding. Depending on parents’ education, statistics or other interpretative information might be more appropriate to present as pictorial representations of chance rather than formal charts and graphs [34]. And for visual imagery of prematurity, congenital anomalies, and even medical technologies, sketch renderings might be easier first images for parents to see than real-life photos. Of course, consideration ought also to be given as to how chosen imagery might influence the relation of family to expectant child. Images have the capacity to personify the fetus as a child [35].

An antenatal consultation as a conversation hopefully culminates in discussion that prepares the hospital staff and expectant parents for what is to come. Media could be used to walk parents through possibilities in the delivery room to possibilities of the NICU stay. Such media could be particularly useful for parents on bed rest, laboring, or otherwise unable to attend actual tours of the NICU before delivery. Serial images of the maturation of a premature infant or clinical milestones of NICU stay could be used to convey a sense of time.

It is clear that good documentation of an antenatal consultation generally means writing down more than a resuscitation plan (chest compressions or no chest compressions; resuscitation medications or no resuscitation medications; and so forth). Possibilities that were discussed should be detailed so they can be reviewed and revisited. Audio recording first conversations between neonatologists and parents has proven useful for promoting information retention for parents [36]. Regardless of the kind of media designed to promote communication of information, it is clear that media must not simplify uncertainty to false convictions or promises to parents that cannot be kept by health professionals and vice versa. There is a clear need for media to assist consultants in patient-charting and communicating antenatal consultation understanding to members of the neonatal team not attendant during the consult. Similarly, there is a potential opportunity for media to support parents’ recording, journaling, or otherwise responding to the informational content of antenatal consultation.

Ideally, provisions need to be made for families to have ongoing access to practitioners so conversations can be revisited. Parents may need time to process information from antenatal consults such that utilizing media that afford self-scheduling for requesting additional consultation might be appropriate. Given that nurses and other non-physician health professionals are significant sources of information for parents in the NICU, designers ought to consider how media might be used to cultivate multidisciplinary interactions [37]. If we consider the antenatal consult as facilitating connections not just with health professionals but also with other NICU families, use of electronic support forums or parent peer support groups might be appropriate. There is likely value for parents to connect with other parents, or at least read NICU family narratives, recognizing that ultimately these other parent experiences might be quite different from their own.

It should be clear that there are limitations to any antenatal consultation related to the sheer volume of information that could be discussed, situational factors such as rapid labor or missing family members, as well as the fact that ultimately when a child is born parents have new experiences in the delivery room and NICU that might change what they need from conversations with hospital staff. Therefore, ultimately an antenatal consultation should be envisioned to be only one of many moments of contact between patient-families and health professionals. Parents might benefit from additional resources (whether it is books or websites) that revisit or provide additional information on an antenatal consultation. Some information clearly is easier to share in a general fashion related to way finding and visitor policies; common medical equipment like incubators and monitors; glossary meanings of common medical terms and acronyms; breastfeeding and nutrition; and so forth.

Our study provides starting points for design research to support antenatal consultation from a relational ethics perspective. Although we reached saturation from analysis of our data, we recognize that the included hospital staff and parent participants do not necessarily represent those who did not attend the focus group sessions. As such, a limitation of our study is the possibility of selection bias. The research nurse only approached parents who were felt by the social worker to not be under psychosocial duress. Correspondingly, participation by hospital staff was on a voluntary basis.

We recognize that our empirical data consist primarily of opinions and perspectives that while being important to inform design are incomplete when considering the wealth of material that could be gained from other research methods. Our study is thus a starting point for future design research on antenatal consultation. For example, the relative importance of the identified themes could be explored. Alternatively, proposed media could be the subject of evidence-based design research [38].

## Conclusions

Antenatal consultations need to be situated in a caring relation to support the emerging relations of baby, parent, and hospital staff through conveying care, understanding, and empathy; creating possibilities for open and genuine conversations; and, fostering the buildings of respect, confidence, and trust. The informational complexity of antenatal consultation warrants thoughtfulness on the part of designers to be sensitive to the range of family experiences, values, and cultures as well as the situational complexities of expecting, delivering, and caring for a child who requires neonatal intensive care. Media must not simplify uncertainty nor erase hope for a positive outcome. Designers also need to be considerate to the desires of the larger medical team, particularly related to consistency, transparency, and validity of the consultation process and any plans that are formulated from it.

While it is valuable to recognize what we hope for in a good antenatal consultation in its current form, it is also important to imagine new possibilities for consultation acknowledging that such possibilities must not disrupt the relational ethics of antenatal consultation. The use of social media, interactive applications, and integrative media that span a family’s journey through the NICU create new possibilities for what can and should be achieved in an antenatal consultation.

From this study it would seem that a health professional’s sensitivity to the expectant family situatedness is at the heart of a good antenatal consultation. It is from such an understanding of a family’s condition and concerns that a practitioner can build with the family a plan for the delivery and care of their child in the NICU. While there may clearly be times that health professionals and parents disagree, ideally shared decision making starting from a ground of common conversational understanding ought to lead in the majority of times to plans that are morally and ethically sound to both professionals and parents.

## Ethics

Permission to conduct this study was obtained from the University of Alberta Health Research Ethics Board and appropriate administrative health authorities.

## Consent

All participants reviewed a study information sheet prior to attending the focus groups. The information contained on the sheet was reviewed prior to starting each focus group concentrating on confidentiality and voluntariness. Informed consent was obtained from all participants. Participants consented to publication of anonymized quotes from the focus group transcriptions.

## Availability of supporting data

Raw focus group data including audio-recordings and transcriptions are unavailable given the need to preserve the anonymity of participants.

## Abbreviations

HCP: healthcare professional
NICU: neonatal intensive care unit
